# A Multi-Objective Compounded Local Mobile Cloud Architecture Using Priority Queues to Process Multiple Jobs

**DOI:** 10.1371/journal.pone.0158491

**Published:** 2016-07-15

**Authors:** Xiaohui Wei, Bingyi Sun, Jiaxu Cui, Gaochao Xu

**Affiliations:** 1 College of Computer Science and Technology, Jilin University, Changchun, China; 2 College of Software, Jilin University, Changchun, China; 3 Symbol Computation and Knowledge Engineer of Ministry of Education, Jilin University, Changchun, China; University of Catania, ITALY

## Abstract

As a result of the greatly increased use of mobile devices, the disadvantages of portable devices have gradually begun to emerge. To solve these problems, the use of mobile cloud computing assisted by cloud data centers has been proposed. However, cloud data centers are always very far from the mobile requesters. In this paper, we propose an improved multi-objective local mobile cloud model: *Compounded Local Mobile Cloud Architecture with Dynamic Priority Queues* (*LMCpri*). This new architecture could briefly store jobs that arrive simultaneously at the cloudlet in different priority positions according to the result of auction processing, and then execute partitioning tasks on capable helpers. In the *Scheduling Module*, NSGA-II is employed as the scheduling algorithm to shorten processing time and decrease requester cost relative to PSO and sequential scheduling. The simulation results show that the number of iteration times that is defined to 30 is the best choice of the system. In addition, comparing with *LMCque*, *LMCpri* is able to effectively accommodate a requester who would like his job to be executed in advance and shorten execution time. Finally, we make a comparing experiment between *LMCpri* and cloud assisting architecture, and the results reveal that *LMCpri* presents a better performance advantage than cloud assisting architecture.

## Introduction

As a result of advances in mobile communication technology, an increasing number of people are using mobile devices. Mobile devices, such as smart phones and tablets, play an important role in daily life. These portable devices provide convenience and pleasure to users and, in doing so, break the restrictions of time and space. Nevertheless, because of inherent hardware constraints, such as low CPU speeds, limited battery capacities and heat dissipation problems, mobile devices are not in a position to perform many computation-intensive tasks [1].

Fortunately, the concept of cloud datacenters provides various solutions to these problems. Because such centers provide abundant computation and storage resources, handheld devices can offload their complex applications to the cloud datacenter for execution by the cloud instead of the mobile devices. For this reason, the concept of mobile cloud computing was proposed [2]. However, because of the long transmission distances from portable devices to cloud data centers, such a mobile cloud model would suffer from longer latency times and weaknesses of wireless networks. In order to solve these problems, two other mobile cloud models have been presented: one model uses a number of nearby mobile devices as helpers to assist in fulfilling the requests of the handheld devices, and the other model uses a cloudlet, which is a nearby server, to fulfill such requests.

In this paper, we propose a novel architecture that replaces the distant cloud using cloudlets and mobile helpers. Part of helpers and cloudlet would be able to provide assistance to requesters in a wireless local area network. To avoid the assistance programming interfering application programs which are running in helpers, we think isolation techniques could be used in the situation. Through isolation techniques, the assistance proceeding is like normal application programs in mobile devices except that it is monitored and isolated from local applications [3]. In order to resolve the problems that arise when multiple jobs arrive at the local cloud almost simultaneously, we introduce queues and priorities. Via the architecture, we attempt to solve the multi-objective scheduling problem that includes minimal execution time and minimal requester cost of a job. Here, the incoming jobs are briefly stored in different priority positions according to the result of auction procedure, and that means in every auction round, the winning job could get the current best position. The modified system is called *Compounded Local Mobile Cloud Architecture with Dynamic Priority Queues* (*LMCpri*). In addition, we focus on scheduling and allocating tasks on helpers in this paper. Because a multi-objective problem is needed to solve, non-dominated sorting genetic algorithm II (NSGA-II) [4] is employed, which uses Pareto dominance principle to achieve comparison of different individuals under distinct objectives. To summarize, our contributions are as follows:

- We propose *LMCpri* to achieve high QoS of requesters, and *LMCpri* satisfies minimum execution time and cost of requesters.
- We concentrate on the scheduling algorithm in *Scheduling Module*, and NSGA-II is chosen as scheduling algorithm. The best number of iteration is 30 via comparisons.
- We evaluate *LMCpri* and *LMCque*, which does not include priority queues, by execution time, and prove that *LMCpri* is better than *LMCque*. Moreover, we obtain the most suitable number of priority queues is six via comparing performance-price ratio.
- *LMCpri* is compared with cloud assisting architecture through *CloudSim* platform, and the result reveals that *LMCpri* is more suitable for the model.

The remainder of this paper is organized as follows. In the following section, we introduce various related works that have been made by researchers. In Section 3, an improved architecture is illustrated in detail. Section 4 presents the experimental results and analyzes of the ameliorating framework. Finally, in Section 5, we conclude the paper and discuss future work.

## Related Work

Mobile cloud computing has served in a large range of domains, including health care [5], electronic commerce [6] and online gaming [7]. Most applications of them are relatively heavy load for mobile devices. To support these applications, researchers have proposed many frameworks that offload heavy-load jobs from portable devices. There are three general mobile cloud computing architectures [8]: (1) augmenting the execution of mobile applications using cloud resources; (2) enabling mobile devices to work collaboratively as cloud resource providers; and (3) extending the access to cloud services to mobile devices.

The first architecture takes advantages of traditional cloud computing into mobile computing. The MAUI offloading architecture attempts to offload fine-grained code to maximize energy savings with a minimum burden from mobile devices on the infrastructure, as opposed to coarse-grained process migration [9]. CloneCloud is a system that uses a combination of static analysis and dynamic profiling to perform partitioning that enables unmodified mobile applications running in an application-level virtual machine to seamlessly offload part of their execution from mobile devices onto device clones operating in a computational cloud [10].

The second mobile cloud computing architecture can be characterized into two types of architectures. One type of representative framework is given in [11] and [12] is an end-to-end model. The requester device is able to discover a nearby optimal portable device; then, the optimal mobile device will help the requester execute the application. The other model relies on the remaining resources of adjacent handheld devices, which would constitute an ad hoc network used to assist the mobile requesters, such as in MobiCloud [13].

Because cloud data centers are always far away from the mobile users, e.g., Amazon’s EC2 infrastructure is located in only six cities worldwide [14], one architecture proposed utilizing a cloudlet [15] that is a trusted, resource-rich computer or cluster of computers that is well-connected to the Internet and is available for use by nearby mobile devices. Although this framework is situated at the virtual machine level, which is a coarse-grained migration, T. Verbelen et al. proposed a component-level migration that is a fine-grained process migration [16]. K.A. Khan *et al*. quantifiably demonstrated advantages of the cloudlet paradigm over its Internet cloud counterpart in supporting the quality of service of real-time application in [17]. However, due to the nature of cloudlet, including managing, scheduling and execution, its resources may not be available to complete all jobs, thus resulting in increased latency.

As for multi-objective task scheduling, meta-heuristic algorithm always presents a desired performance, such as multi-objective genetic algorithm. WBGA [18] was proposed by Hajela P. et al., and it settles different weight to different objective functions. Mostly, the sum of these weights is 1. Although the advantage of the method is convenient to realize, the disadvantage of the method is the choice of weight, as it needs plenty of experiment. Srinivas N. et al. proposed NSGA [19], and its fitness assignment is based on non-domination sorting. In order to preserve diversity in the population, they introduce the biological concept of niche, and then properly increase the fitness of the individuals which are in the area of sparse density. The same as WBGA, NAGA is required to define a niche size parameter artificially. So as to avoid artificial operation and minimize handling time, Deb K. et al. proposed NAGA-II that it uses the distance between the (*x-1*) and (*x+1*) in the same Pareto front on every objective function to measure the density, where *x* replaces every individual [4].

## Architecture and Scheduling Algorithm

### 1. Compounded Local Mobile Cloud Architecture

**Fig 1:**
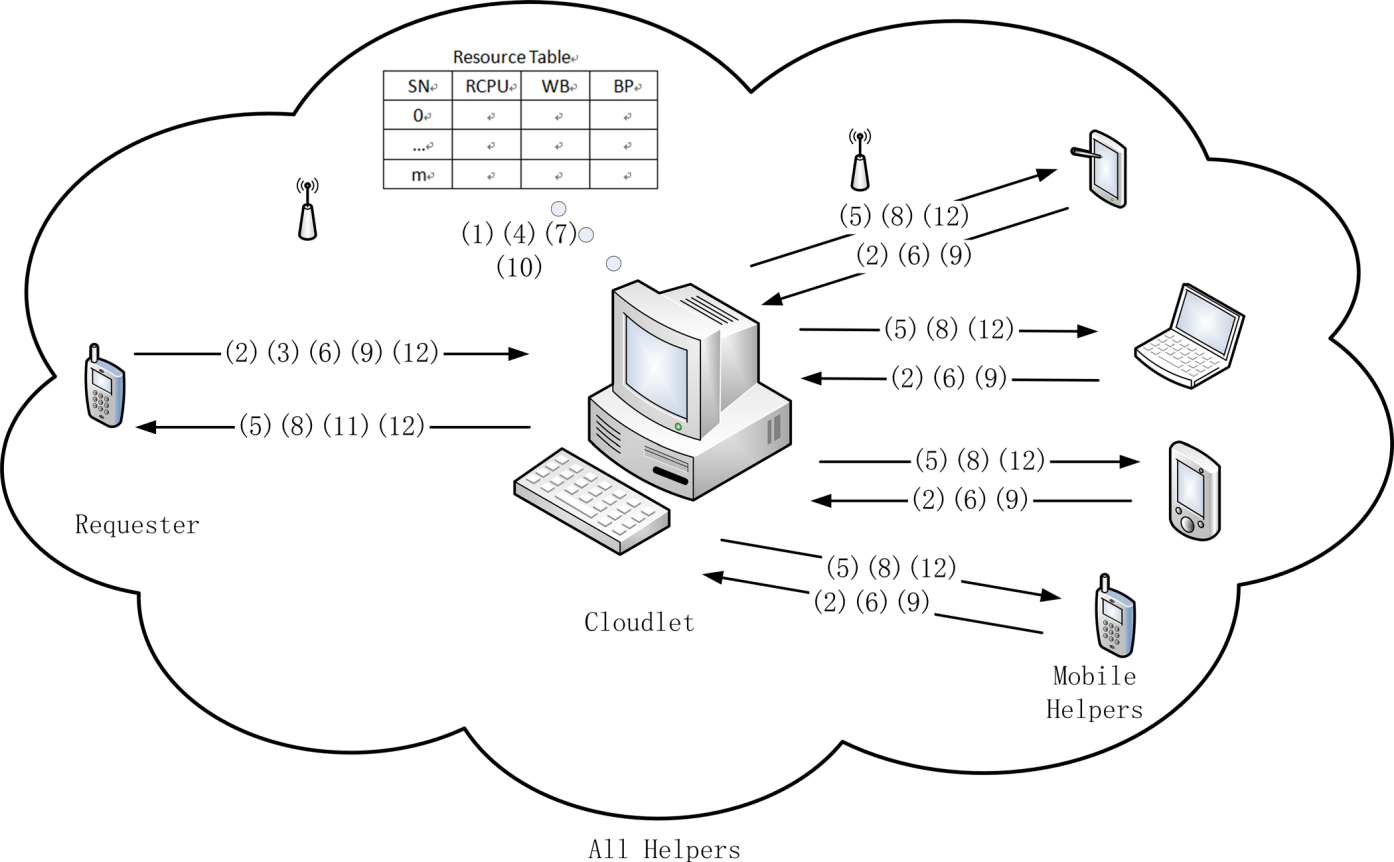
Compounded local mobile cloud architecture. This model is used to present the execution process of *CLMCA*.

In order to improve real-time performance and make requesters experience high quality of services (QoS), the new architecture should decrease latency and cost. Based on these characteristics and to address the disadvantages of existing architectures which are presented in related works, we introduce a cloudlet and mobile helpers that have relatively sufficient resources into the new architecture. The new architecture is called *Compounded Local Mobile Cloud Architecture* (*CLMCA*) and is described in Fig 1.

According to Fig 1, the operation of *CLMCA* can be generalized into the following twelve steps:

1. Cloudlet monitors the local area network.
2. Mobile helpers send their own information to the cloudlet regularly, and the period is Δt. The collection module in the cloudlet collects information, which includes the available CPU power (*RCPU*), available battery power (*BP*), wireless bandwidth (*WB*) and assigned sequence numbers (*SN*), which range from 1 to *m*, from each mobile helper (the serial number of the cloudlet is 0). We suppose that there are *m* handheld device helpers. Users of mobile helpers could set the *RCPU*, *WB* and *BP* by themselves according to current using situation. After collecting information, cloudlet maintains its resource table, which is utilized to store information of helpers.
3. Requester offloads a job that it cannot execute by itself to the cloudlet through wireless network and requests service.
4. The cloudlet partitions the job and requires a heuristic algorithm for scheduling. The scheduling method should make the subtasks faster and more economically, according to collected information.
5. Cloudlet sends a helpful packet to all selected helpers in a broadcasting way within its local area network.
6. If the helper does not leave the area, it would return a response and its available parameters to cloudlet.
7. Cloudlet updates recourse table and statistics status of live helpers within a waiting time, *deadTime*. If all helpers, which are chosen to assist requester, response to cloudlet, it will enter the next step. If any selected helper does not reply until *deadTime*, cloudlet goes to have to return to step (4).
8. Cloudlet dispatches tasks to helpers.
9. The working helpers return findings and performance parameters to cloudlet.
10. The work of the cloudlet is to integrate these data and the costs, which the requester should pay to all helpers through the wireless network, and meanwhile, update recourse table. If any results are no longer returned within the deadline, *outTime*, cloudlet will rank helpers that are in the latest resource table in descending order, and then send uncompleted tasks to superior helpers again.
11. Cloudlet returns the final result and the deserved reward to requester.
12. Requester pays for the service to helpers in the local mobile cloud through cloudlet.

While there is a problem that is when many requesters send their help requests almost simultaneously, the original *CLMCA* is not able to store these jobs. To solve the problem, queues are added to the architecture.

### 2. Compounded Local Mobile Cloud Architecture with Queues

**Fig 2:**
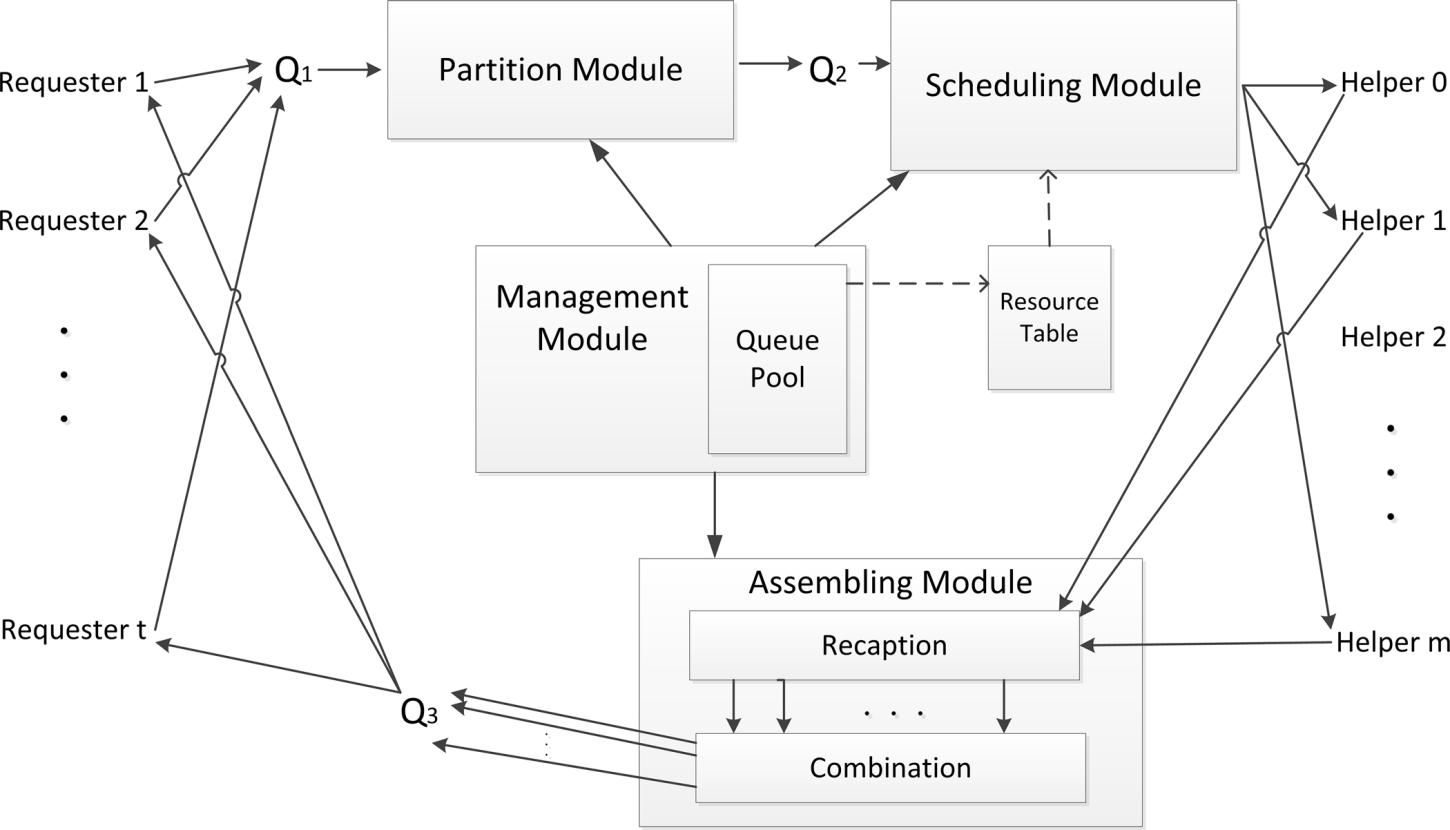
*LMCque*: Construct a Queue Pool to briefly store incoming jobs. Q1, Q2, Q3 are queues, which are used to stash jobs or tasks or results. Resource table is used to store collected information about helpers. All of them are dominated by Management Module.

The new system is called *Compounded Local Mobile Cloud Architecture with Queues* (*LMCque*), and the framework is shown in Fig 2.

To establish the modified system, we propose four hypotheses in this paper. The first hypothesis is that the wireless connections between portable devices and the cloudlet are sufficient. The second hypothesis is that not only the cloudlet but also all handheld devices must install the collecting equipment to collect the necessary information from itself. The third hypothesis is that Q1, Q2 and Q3 have the ability to store all of the incoming jobs at any time point. The last hypothesis is that during the time of updating recourse table, none of the collected data change.

As Fig 2 shows, the architecture consists of *Management Module*, *Partition Module*, *Scheduling Module* and *Assembling Module*. We use pseudo code to display core functions of these modules. In initial situation, all modules listen to message continually and wait for input data.

*Management Module* is used to maintain resource table and administrate *Queue Pool* in *LMCque*. The *Queue Pool*, which consists of Q1, Q2, and Q3, is a newly introduced storage cell. So as to achieve effectiveness of a buffer pool, the *Queue Pool* is used to provisionally store the arriving and leaving jobs which are from mobile requesters and *Assembling Module*, respectively. Q_1_ takes charge of storing intact jobs received from requesters when *Partition Module* is in busy. Q_2_ is responsible for stashing tasks which are partitioned from jobs, and waits for scheduling operations. Q_3_ is in charge of storing the final result of each job before returning to requesters.

After leaving Q_1_, job enters into *Partition Module*. The goal of the *Partition Module* is to partition a job, which is in the waiting queue, into multiple tasks and then registers the size of sending instruction transmissions (*IT*^*s*^), for which the unit is 1 MB, and the calculation amount (*CA*), for which the unit is 1 MIPS, of every task. Therefore, a suitable partitioning method should be adopted. After that, we dispose the partitioned tasks into Q2, and then delete the job from Q1. Finally, the split result is returned to ensure a successful partition.

Before the tasks are scheduled, the *Management Module* collects information of all helpers again to ensure that they have the most recent data and stores them in the resource table. Following the resource table, *Scheduling Module* executes the scheduling algorithm. The duty of *Scheduling Module* is to dispatch all these tasks to helpers, which have sufficient resources to execute the tasks according to the resource table, and push tasks into a waiting queue *taskQ* of corresponding helpers. Within the procedure, we can obtain CPU power dissipation (*CP*), sending power dissipation (*SP*) and receiving power dissipation (*RP*) through the Wi-Fi Radio [20].

If a mobile phone is selected as a helper and it is not busy, it will check its *taskQ* to see whether it is empty. If there is at least one task in *taskQ*, the task will be handled by the helper and then popped from *taskQ*.

Finally, cloudlet retrieves findings of tasks from all the helpers, assembles the results, and sends it to Q_3_ through *Assembling Module*. In *Assembling Module*, the core function is *startAssembly()* and the module maintains two vectors named *RcvMsgs* and *tempRcvMsgSet*. The vector *tempRcvMsgSet* contains a set of consequences of tasks. *RcvMsgs* is a vector set, which stores vectors constructed by results of tasks. When *tempRcvMsgSet* is not empty, a result *taskRcvMsg* will be taken out from it. If there is a vector related to a job (*taskRcvMsg* is a consequence of a task which belongs to the job) in *RcvMsgs*, and then *taskRcvMsg* will be added into the vector of *RcvMsgs*. Otherwise, Add *taskRcvMsg* to a new vector and add the new vector to *RcvMsgs*. If all results of the job of *taskRcvMsg* have been collected, then clear up all of the results of this job to *filnalResult* and push *filnalResult* into Q3. After handling *taskRcvMsg*, it will be popped from *tempRcvMsgSet*.

However, when some jobs are urgent and the requester would like to pay more money to complete it early, a single Q_1_ in the Queue Pool cannot achieve this function. Hence, we apply the idea of setting jobs to dynamic different priority queues.

### 3. Compounded Local Mobile Cloud Architecture with Dynamic Priority Queues

**Fig 3:**
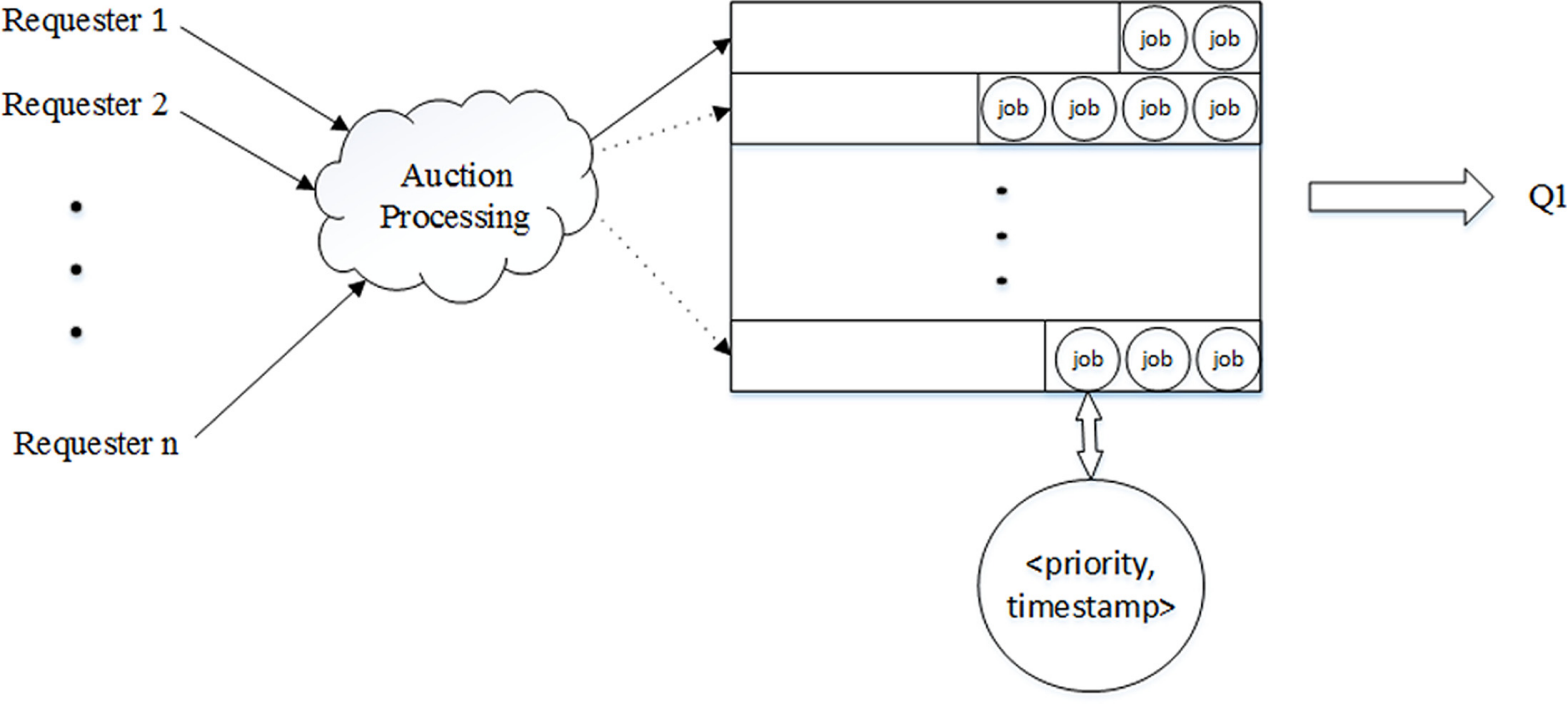
A detailed description of dynamic priority queue. Through auction processing, the winning job obtains the current best position, and enters into corresponding priority queue with timestamp. Then, the job waits for being scanned and inputted in Q_1_.

To resolve the problem created by only one waiting job queue, a set of dynamic different priority queues are set before Q_1_. The modified framework is named *Compounded Local Mobile Cloud Architecture with Dynamic Priority Queues* (*LMCpri*), and its constructed specification is presented in Fig 3.

**Fig 4:**
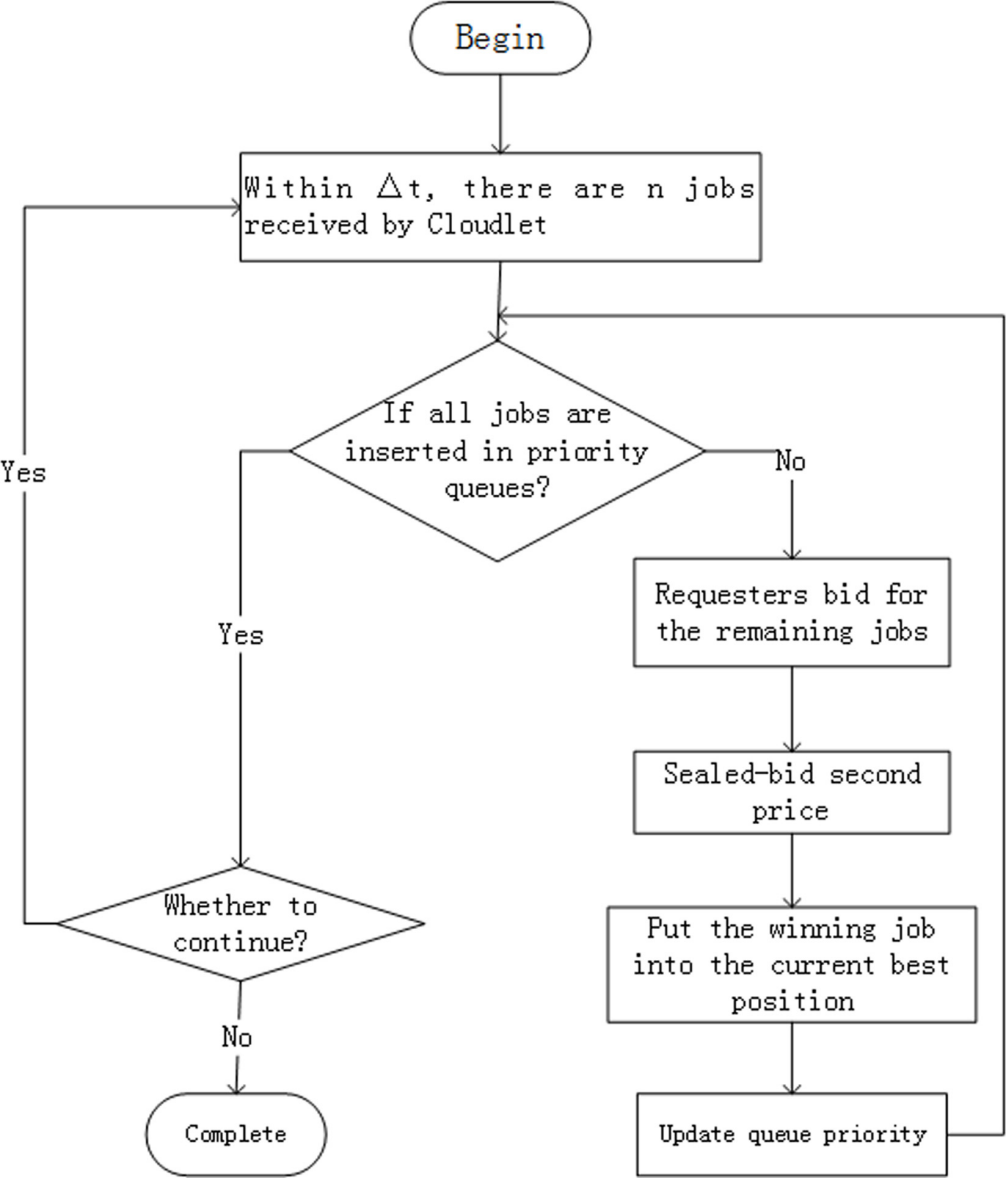
A flowchart of priority processing.

As Fig 3 shows, all the jobs are auctioned according to the sealed-bid second price [21]. Because the sealed-bid second price is a weak dominant strategy, it is the best choice for all requesters to bid at the same price as their valuation. By this method, we ensure that requesters tell the truth, and there is no inflating price situation. The winning job is inserted into the current best position in a greedy way and the best position is selected in terms of the following formulation:

$$vp=\frac{jn}{\sum_{h=1}et},$$ (1)

in which *vp* means processing velocity of the priority queue currently. Moreover, *jn* and ∑_*h* = 1_ *et* are the number and the sum of estimated time of jobs which are receiving by the queue, respectively. Initially, the priorities of these priority queues are set randomly, and *vp* is equal to ∞. After inserting a job, *vp* is smaller than ∞, so the queue is not the current best position. The estimated time (*et*) of a job could compute according to formulation (2):

$$et=\frac{JIT_{s}}{\sum_{i=0}^{m}WB_{i}}+\frac{JIT_{R}}{\sum_{i=0}^{m}WB_{i}}+\frac{JCA}{\sum_{i=0}^{m}RCPU_{i}},$$ (2)

where *m* is the sequence number of helpers and we will describe it particularly in next section. *JIT*_*s*_ and *JIT*_*R*_ are data gross of the requester sending and receiving, respectively, and *JCA* is calculation quantity of the job. After entering into the current best position, the job is added timestamp to ensure that only a job would be sent into Q_1_ even there are two jobs with the same priority at a certain time. Through this method, if a job transmits request in the second △t, but bids at a high price, it may be executed earlier than some jobs that arrive in the first △t. On the output end of priority queues, we scan them in sequence, and choose the job with the highest priority. If there are two jobs with the same priority, the job having a small timestamp is going to be sent into Q1 first. The dynamic priority processing flowchart is presented in Fig 4.

After leaving priority queues and waiting queues, the job is moved in *Partition Module*, *Scheduling Module*, and *Assembling Module* in sequence, the same as *LMCque*. The *Scheduling Module* is discussed mainly in the paper, therefore, multi-objective scheduling method is presented in the next section.

### 4. Multi-Objective Scheduling Problem Formulation

This paper shows two different objectives which are minimizing requester cost and minimizing execution time in *LMCpri* system. On the one hand, every processor has its own computing capacity, thus varied processing time and power consumption are exhibited within a set of processors, even if each processor completes the same task. On the other hand, because there is a distinct amount of wireless bandwidth available for every helper, the transmission time and the transmission costs differ with each other. Since the measurement unit of the size of the transmission task is *1MB*, while the unit of bandwidth is *bps*, in order to uniform units, and make the formulation seem convenient, *WB* is multiplied by a constant *0*.*128* before it is introduced in formulations. Here, three assumptions have been taken. First, a job is partitioned into *n* tasks; there are (*m+1*) helpers, which consist of *m* mobile helpers and the cloudlet, and *n* is not smaller than (*m+1*). Second, these partitioning tasks run independently, and all of the helpers are heterogeneous. Therefore, the multi-objective task scheduling problem can be proposed as mapping *n* tasks onto (*m+1*) helpers to minimize the total execution time and the requester cost. Every individual would include an n-dimension solution vector, and the *j-th* element of this vector is the *SN* of the helper that is chosen to perform the *j-th* task. Thirdly, because the framework is a small business model, and it is suitable in a local area network, we do not consider about blocking or delaying situations during the transfer time.

If one helper wants to assist requesters to process tasks, the power that it can supply should be greater than the power consumed by the tasks. Based on the collected information, we present the limiting condition as follows:

$$\sum_{z}\left(\frac{{CA}_{z}}{{RCPU}_{i}}\times CP+\frac{{IT}_{z}^{R}}{{WB}_{i}}\times RP+\frac{{IT}_{z}^{S}}{{WB}_{i}}\times SP\right)\leq {BP}_{i},$$

z∈{1,2,⋯,n},i∈{0,1,2,⋯,m}. (3)

This means that a helper whose *SN* is equal to *i* can accomplish the *z-th* task, which may include more than one task, and that the total consumed power of these tasks is less than the power that the *i-th* helper can offer. Under these conditions, we hypothesize that all n tasks can be completed by the (*m+1*) helpers. Thus, we introduce a metric denotation *θ* to register the number of executed tasks; according to the problem description, the sum of the components of *θ* should be equal to *n*. The definition of *θ* is

$$\theta =\sum_{j=1}^{n}\sum_{i=0}^{m}\gamma_{j}^{i}=n,\;j\in \left\{1,2,\cdots ,n\right\},\;i\in \left\{0,1,2,\cdots ,m\right\}.$$ (4)

In this equation, $\gamma_{j}^{i}$ denotes that the *j-th* task could be executed by the *i-th* helper, and its definition is

$$\gamma_{j}^{i}=\{\begin{array}{c} 1,\;\text{the}\;i\text{-}th\;\text{helper could execute the}\;j\text{-}th\;\text{task},\\ 0,\;\text{the}\;i\text{-}th\;\text{helper could not execute the}\;j\text{-}th\;\text{task}. \end{array}$$ (5)

In this paper, we want to solve a multi-objective task scheduling problem in terms of the minimal execution time and the minimal requester cost; thus, the objective function should be discussed. The first goal is to minimize execution time, which includes the helper processing time and the transmission time, both of which depend on the *CA*, *RCPU* and on the *IT*^*S*^, *IT*^*R*^, *WB*, respectively. According to [22], the execution time function can be written as

$$T=Max\;\left(\sum_{j=1}^{n}\left(\frac{CA_{j}}{RCPU_{i}}\times \gamma_{j}^{i}+\frac{IT_{j}^{R}}{WB_{i}}\times \gamma_{j}^{i}+\frac{IT_{j}^{S}}{WB_{i}}\times \gamma_{j}^{i}\right)\right),\;i\in \left\{0,1,\cdots ,m\right\}.$$ (6)

The second objective is to minimize the cost of all requesters, which includes the power consumption of the helpers and the bandwidth cost of transmission, both of which in turn are dependent on *CA*, *IT*^*R*^ and *IT*^*S*^, respectively. Here, we define *C*_*Pro*_ as the processing cost, *C*_*OUT*_ as the sending cost and *C*_*IN*_ as the receiving cost. Therefore, the requester cost function of the jobs in no priority queue is as follows:

$$C_{base}=\sum_{i=0}^{m}\left(\sum_{j=1}^{n}\left(\frac{CA_{j}}{RCPU_{i}}\times C_{Pro}\times \gamma_{j}^{i}+\frac{IT_{j}^{R}}{WB_{i}}\times C_{IN}\times \gamma_{j}^{i}+\frac{IT_{j}^{S}}{WB_{i}}\times C_{OUT}\times \gamma_{j}^{i}\right)\right).$$ (7)

When considering about priority, different extra cost should be added to the requesters’ cost. According to sealed-bid second price [21], the winner in each auction round should pay the second high bid price to obtain the best position of priority queues, and we use *C*_*add*_ to present extra cost. Therefore, the total cost could be calculated, which is

$$C_{total}=C_{base}+C_{add}.$$ (8)

Based on these objective functions, the fitness functions can be written as follows:

$$\{\begin{array}{c} T=Max\;\left(\sum_{j=1}^{n}\left(\frac{CA_{j}}{RCPU_{i}}\times \gamma_{j}^{i}+\frac{IT_{j}^{R}}{WB_{i}}\times \gamma_{j}^{i}+\frac{IT_{j}^{S}}{WB_{i}}\times \gamma_{j}^{i}\right)\right),\\ C_{base}=\sum_{i=0}^{m}\left(\sum_{j=1}^{n}\left(\frac{CA_{j}}{RCPU_{i}}\times C_{Pro}\times \gamma_{j}^{i}+\frac{IT_{j}^{R}}{WB_{i}}\times C_{IN}\times \gamma_{j}^{i}+\frac{IT_{j}^{S}}{WB_{i}}\times C_{OUT}\times \gamma_{j}^{i}\right)\right),\\ C_{total}=C_{base}+C_{add}, \end{array}$$ (9)

which should satisfy the constrains:

{∑z(CAzRCPUi×CP+ITzRWBi×RP+ITzSWBi×SP)≤BPi,θ=∑j=1n∑i=0mγji=n.z∈{1,2,⋯,n},j∈{1,2,⋯,n},i∈{0,1,2,⋯,m}. (10)

### 5. Task Scheduling using NSGA-II Algorithm

#### 5.1 Selection Operator

**Fig 5:**
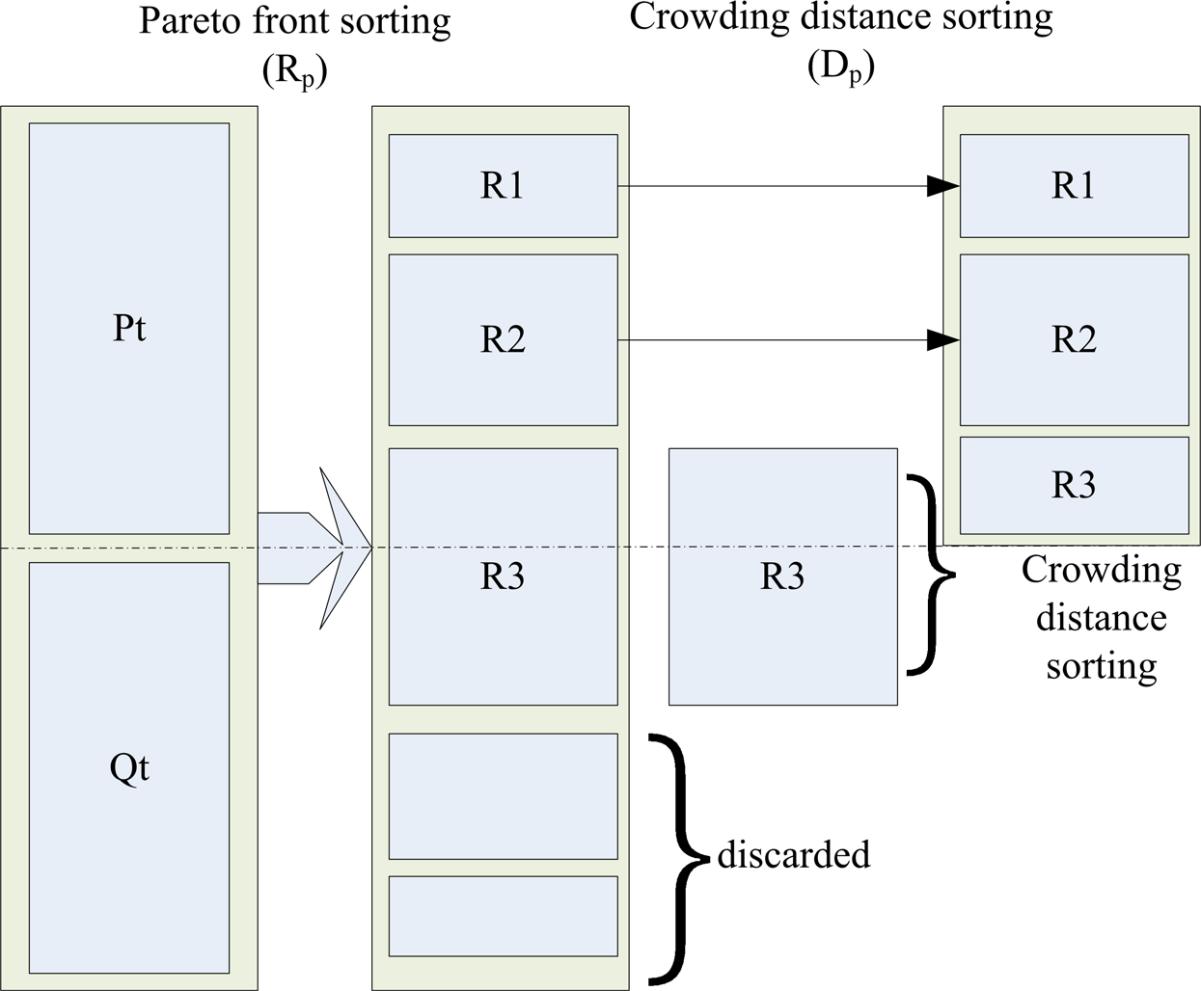
Selection operator of NSGA-II.

Efficient scheduling algorithm is able to increase the searching speed and avoid falling into local optimal early. Therefore, NSGA-II is designated as scheduling algorithm. According to NSGA-II [4], we have two fitness values, *R*_*p*_ and *D*_*p*_. *R*_*p*_ means the front number of *p*, and *D*_*p*_ displays the degree of looseness of the location of *p*. Due to the different significance of these two fitness values, a partial relationship is used in selection operator. The partial relationship ${≺}_{R_{p,}D_{p}}$ is like this:

$$\begin{array}{l} \mu {≺}_{R_{p,}D_{p}}\upsilon \;\text{if}\;\left(\mu_{R_{p}}<\upsilon_{R_{p}}\right)\\ \;\text{or}\;\left(\mu_{R_{p}}=\upsilon_{R_{p}}\right)\;\text{and}\;\left(\mu_{D_{p}}>\upsilon_{D_{p}}\right), \end{array}$$ (11)

where *μ* and *ν* are different individuals. Through this operation, we get a new generation population, in which the individuals possess a smaller R_p_ or a larger D_p_ when they have the same R_p_. Just as Fig 5 shows:

Pt and Qt are used to represent the parent generation and the offspring generation, respectively. When choose appropriate D_p_, in order to increase the calculation speed, a tournament selection operator is employed.

#### 5.2 Crossing Operator and Mutation Operator

Crossing and mutation operators are used to generate more diverse individuals. In this paper, adaptive linear crossing operator is employed. It not only improves the global convergence at the beginning and the local convergence at the ending of processing, but also protects diversity of the population as far as possible. $X_{n}^{t}$ and $X_{m}^{t}$ represent individual *n* and individual *m* in the *t-th* generation respectively. By the linear crossing operator, their offspring $X_{n}^{t+1}$ and $X_{m}^{t+1}$ can be calculated as follows:

$$\{\begin{array}{c} X_{n}^{t+1}=\alpha X_{n}^{t}+\left(1-\alpha \right)X_{m}^{t}\\ X_{m}^{t+1}=\left(1-\alpha \right)X_{n}^{t}+\alpha X_{m}^{t} \end{array},$$ (12)

where *α* is a crossing probability and it is not a constant, since it is denoted according to the fitness value *R*_*p*_, just as follows:

$$\alpha =\frac{m_{R_{p}}}{\left(m_{R_{p}}+n_{R_{p}}\right)}.$$ (13)

As for the mutation operator, a fixed mutation probability *β* is chosen. If the random probability of an element is less than *β*, it will trigger a mutation operator.

After selection operator, crossing operator and mutation operator, the next generation could be obtained, and the number of iteration is *t*. If *t* has not arrived at the max iterations, the procedure will enter into the next iteration, otherwise, an optimal result will be selected randomly from the first front.

## Simulation and Evaluation

### 1. Experimental environment and methodology

We employ *Lenovo ThinkCentre M8400t-N000* installed *32-bit Ubuntu 14*.*04 LTS* to achieve the simulation. The programming language uses C++ and Java. The architecture is implemented by C++ programming language, and we use *vector container* to achieve FIFO function of queues in the architecture. Java is utilized to simulate cloud center through a platform, CloudSim [23].

We consider the real-time performance of the architecture, so jobs which are chosen should take up lots of CPU performance and have a fast processing speed, for example, quick sort on data stream, and data stream, which is composed of natural numbers, is split randomly. In the experiments, the population size (*Pop*), max iteration and mutation probability (*β*) are set at 20, 100 and 0.05. Moreover, we want to explain the architecture by simulation, every job is generated randomly according to size and complexity, so they are selected from intervals [10, 200] (MB) and [500, 5000] (MI) at random, respectively. In order to measure the scalability of our architecture, we set helper number in {4, 6, 8, 12, 16, 20} and task number in {16, 32, 64}. In addition, the helpers’ parameters which include *RCPU*, *BP* and *WB* are randomly chosen from three intervals that are [10, 750] (MIPS), [130,260] (mAh) and {4, 5, 6, 7, 8} (Mbps), respectively. We suppose that the status of helpers do not change within 60s and it is expressed by Δt. The parameters contained by the framework are *deadTime* and *outTime* that are assigned at 4s and 30s, and *C*_*add*_ is randomly selected from interval [0.1, 0.5].

In the following section, the *LMCpri* is evaluated using simulation experiments, which is composed of three parts. First, we compare NSGA-II with PSO and sequential scheduling, via requester cost and total execution time, to present that NSGA-II is the best choice. Moreover, we search for suitable iteration times of task dispatching using the NSGA-II algorithm in our system through different number tasks and helpers to make a determining on a finer scale. Secondly, *LMCpri* is compared with *LMCque* when more than one job arrive simultaneously to illustrate the advantages of *LMCpri*. Thirdly, we make a comparison between the proposed architecture and the cloud assisting architecture that has been introduced by other papers. In order to guarantee the accuracy of data result, we run every experiment 30 times, and then record average values.

### 2. Experiments on Scheduling Selection

In this paper, the NSGA-II algorithm is used as the task scheduling decision method. To show that NSGA-II is a better choice for this special application, we compare it with Particle Swam Optimization (PSO) [24] and sequential scheduling, based on execution time and cost of the requester. The specific parameters of PSO are c_1_ = c_2_ = 2.0, *v*_*max*_ *=* 4.0, *v*_*min*_ = -4.0, x_max_ = 4.0 and x_min_ = 0, and the fitness function could be obtained from (6) and (8), that is

$$\text{fitness value}=0.7\times \text{T}+0.3\times C_{total}.$$ (14)

Moreover, in order to shorten convergence time of NSGA-II algorithm, we try to select an appropriate iteration times for this particular system. In this set of experiments, the same job is used because of the necessity of fixing variant. In addition, we do not set priority for the job, and that means there no extra expenditure. So as to prove scalability of the architecture, the job is partitioned into 16, 32 and 64 tasks, and these tasks are executed on four, six, eight, twelve, sixteen, and twenty helpers, respectively. Since we have supposed that the task number is more than the helper number, we do not consider the situation that 16 tasks are scheduled to 20 helpers.

#### 2.1 The Requester Cost of NSGA-II, PSO Algorithm and sequential scheduling

**Fig 6:**
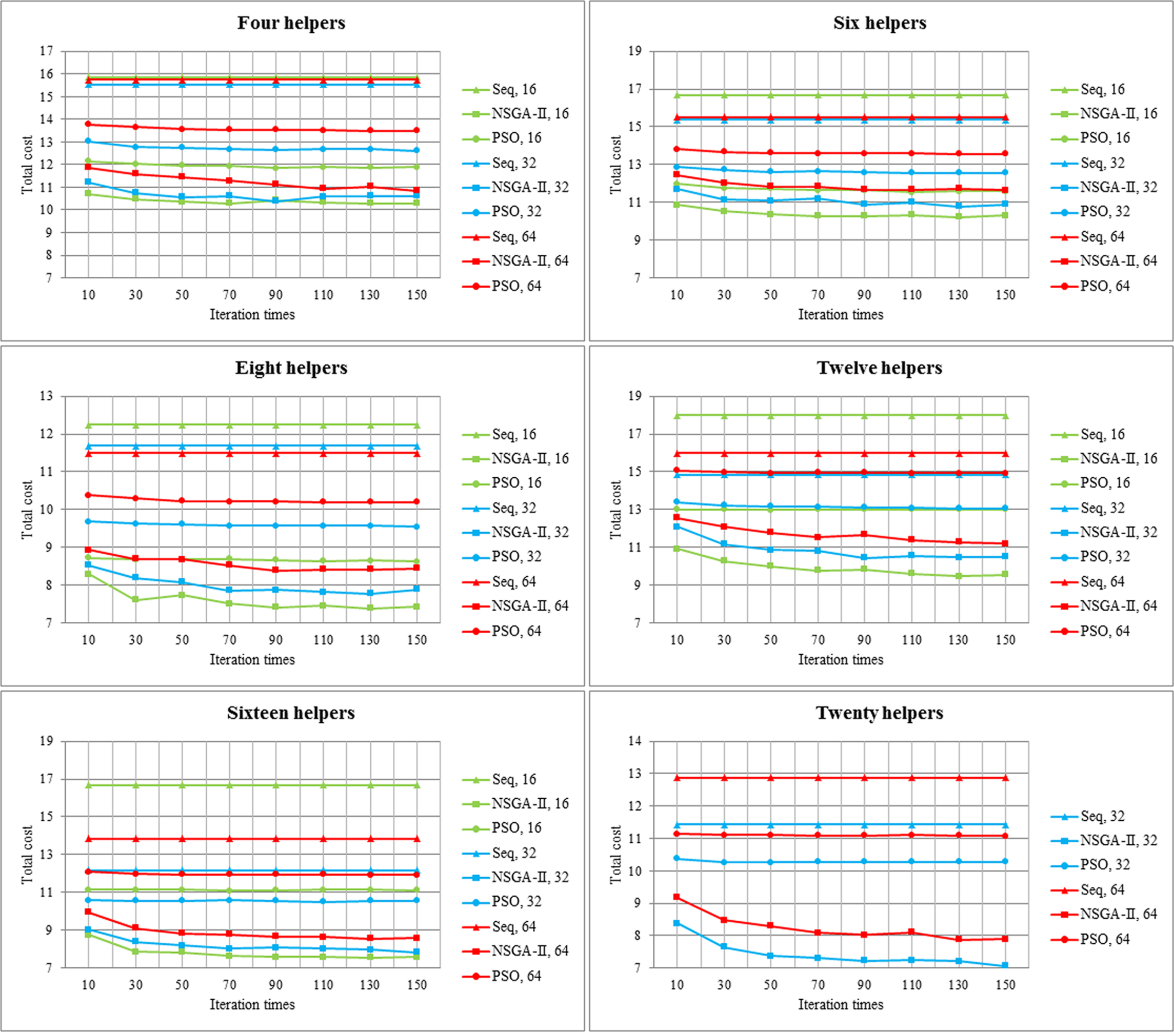
Total requester cost with a varying number of helpers and tasks. The x-axis and y-axis represent iteration times and the total cost, respectively. Every line graph reveals a number of helpers. We use triangle, square, and circle to denote sequential scheduling, NSGA-II and PSO, respectively. Green, blue and red show different task numbers respectively.

Because users in different countries use different monetary systems, the cost presented in this paper is a fiducial comparable value, and the actual expense should be determined according to the factual situations. In this group of experiments, the same job is employed by different experiments. According to reference [20], we can define C_Pro_, C_IN_ and C_OUT_ in Table 1, and the relationship among total requester cost, the number of helpers and tasks is illustrated in Fig 6.

**Table 1:** Power consumption of requester cost items.

Cost item	Average cost
Processing cost (C_Pro_)	0.7
Receiving cost (C_IN_)	0.2
Sending cost (C_OUT_)	0.3

As Fig 6 displays, NSGA-II brings the least total cost compared with PSO and sequential scheduling, and with the increasing number of helpers, the advantage of NSGA-II is shown more clearly. The reason for this phenomenon is that tasks have more choice about choosing which helper to process them, and NSGA-II has the ability to find more accurate global optimal solutions than other scheduling algorithms through Pareto dominance. In addition, whatever the scheduling algorithm, total cost reduces with decreasing number of tasks. That is explained by the fact that it also inevitably lengthens communication time, even though a fine-grained task could be dispatched to a helper which owns stronger task processing capability. Thirdly, with increasing of iteration times, the total cost reduces when task numbers and helper numbers are fixed, and this is because of the convergence of scheduling algorithm.

#### 2.2 The Total Execution Time of NSGA-II, PSO Algorithm and sequential scheduling

When the job is handled on twelve and sixteen helpers by sequential scheduling, it takes 10.1s and 6.1s to complete sixteen tasks, so they spend longer time than other measurement groups. Therefore, we do not present the sequential scheduling time for 16 tasks that are processed on 12 or 16 helpers.

**Fig 7:**
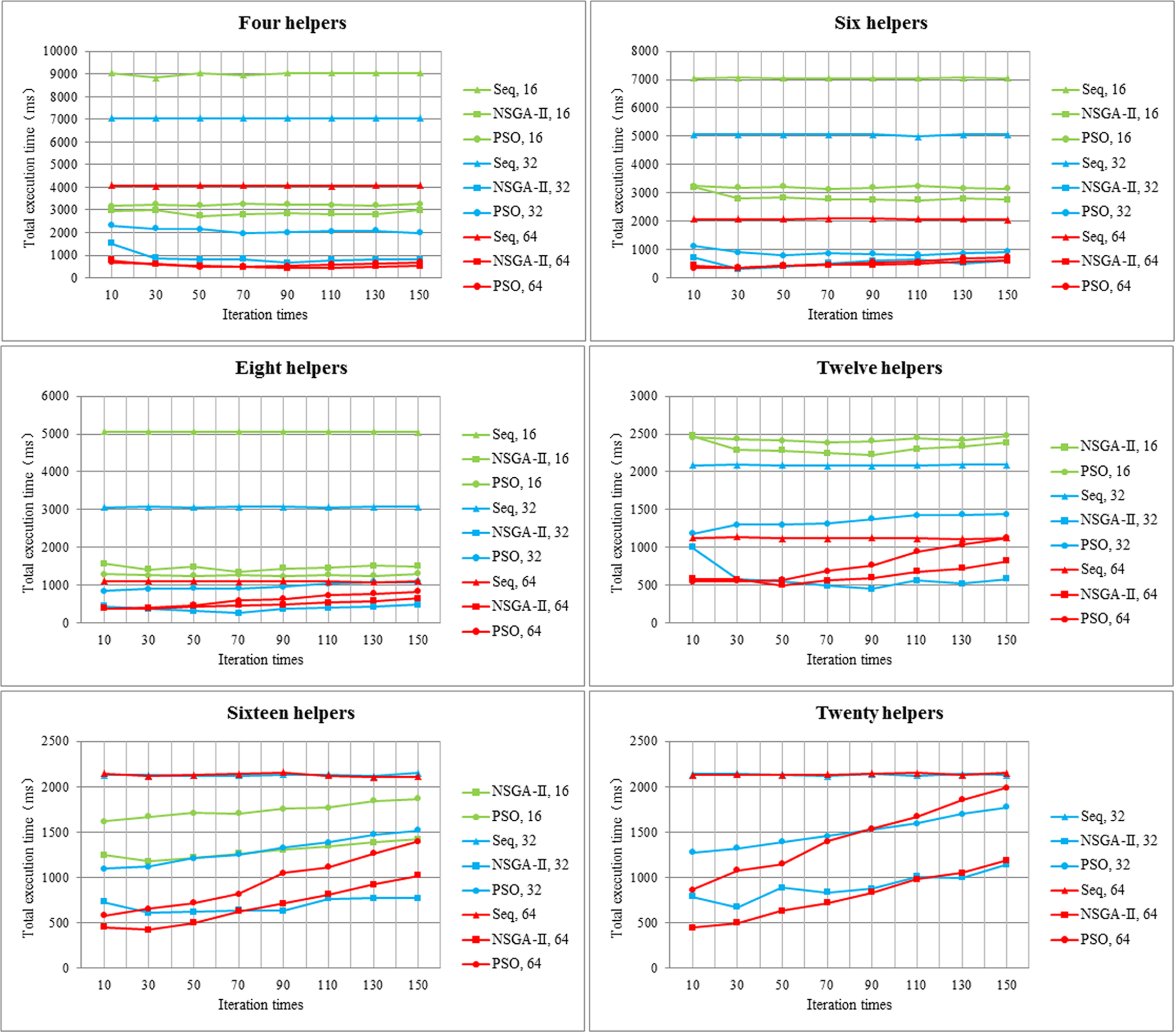
Total execution time with different number of tasks, helpers. The x-axis and y-axis represents iteration times and total execution time, respectively. Every line graph represents a number of helpers. We use triangle, square, and circle to denote sequential scheduling, NSGA-II and PSO, respectively. Green, blue and red show different task numbers respectively.

From Fig 7, we could find that regardless of helper number and task number, NSGA-II always presents the shortest total execution time among the three scheduling algorithms, which uses within 4000ms when there are 16 tasks, and within 2000ms at 32 or 64 tasks. The second one is PSO, and the last one is sequential scheduling. The reason for this phenomenon is that though NSGA-II algorithm spends longer time than PSO in scheduling process, but it can avoid locally optimal solution effectively and tries it best finding the most excellent solution in global searching space, and so it reduces processing time of helpers. In addition, we surprisingly discover that with the increasing number of tasks, NSGA-II does not decrease execution time obviously, and when a job is partitioned into 32 tasks, it reveals a better performance. This may be due to incremental communication time within processors. Though helpers implement tasks during shorter processing time, it takes a long time for transferring. Therefore, 64 tasks do not show a result as expectation. However, with the increasing of helpers, total execution time decreases dramatically.

So as to reduce unnecessary scheduling time, we try to select the most suitable iteration times, and it will be shown in the next section.

#### 2.3 Iteration Times of NSGA-II Algorithm

We find that the total execution time takes on a stable tendency with increasing iteration times. Therefore, for purpose of cutting down execution time, we try to reduce iteration time.

**Fig 8:**
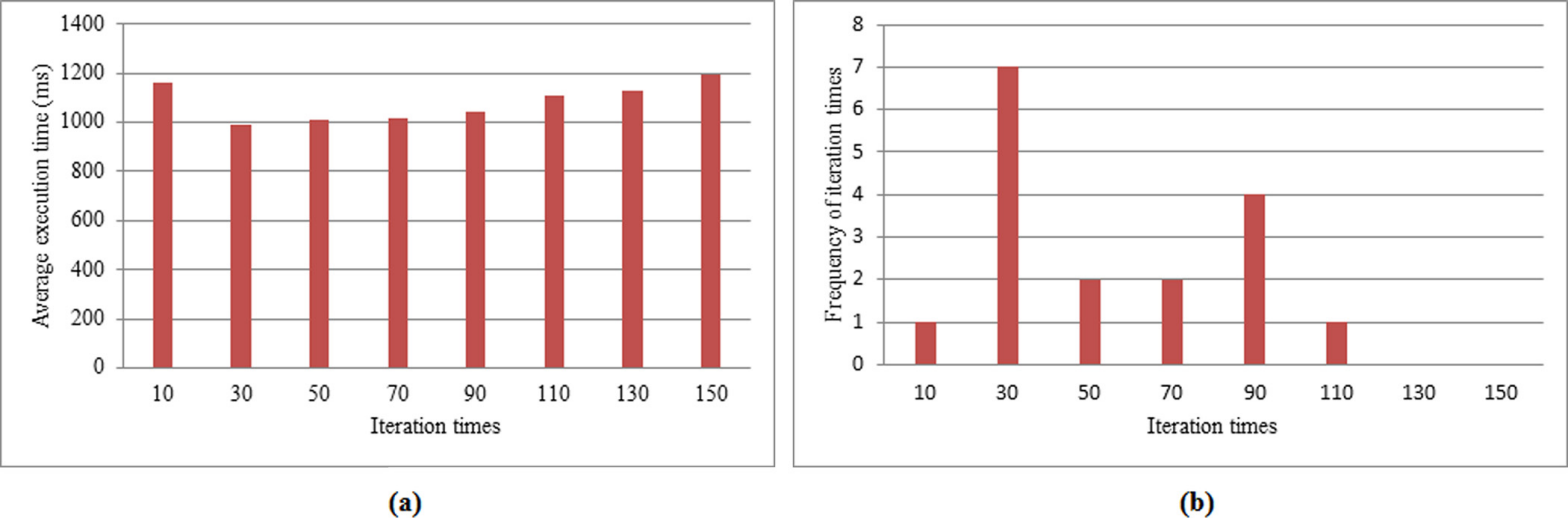
Selecting iteration times of NSGA-II in LMCque and LMCpri. **(a). Average value of execution time of different iteration times.** Different iteration times are exhibited in x-axis, and y-axis presents average value of execution time. **(b). Frequency of iteration times.** Different iteration times are displayed in x-axis, and y-axis presents the frequency of iteration times.

As Fig 8(A) shows, average time drops markedly when the number of iteration times declines from 10 to 30, and it ascends gradually with increasing iteration times from 30 to 150. When the iteration times arrive at 150, average execution time exceeds the time that the number of iterations is 10. So iteration times are 30, the average execution time is shortest, which is less than 1s. From Fig 8(B), it apparently presents that as iteration times reach 30, the frequency of iteration times of the shortest execution time is the maximum value of seven.

The number of iterations equaling to 30 presents the best execution time both in Figs 8(A) and 7(B), because of the strong convergence and global searching capability of NSGA-II. In the following experiments, we will choose the number of iterations as 30 to decrease execution time.

### 3. Evaluation of Compounded Local Mobile Cloud Architecture with Dynamic Priority Queues

In this section, we are going to evaluate *LMCpri*, according to the defined performance-price ratio, and the formula of performance-price ratio is as following:

$$performance\text{-}price\;ratio=\frac{lifeTime-Time}{Cost},$$ (15)

where *lifeTime* represents the longest waiting time of requesters, and we set it at 180s. *Time* and *Cost* stand for the total execution time and the total expense of a job, respectively. Firstly, we want to choose a suitable number for priority queues. Then, we would like to prove *LMCpri* is better than *LMCque* via comparing execution time.

#### 3.1 The number of priority queues

**Fig 9:**
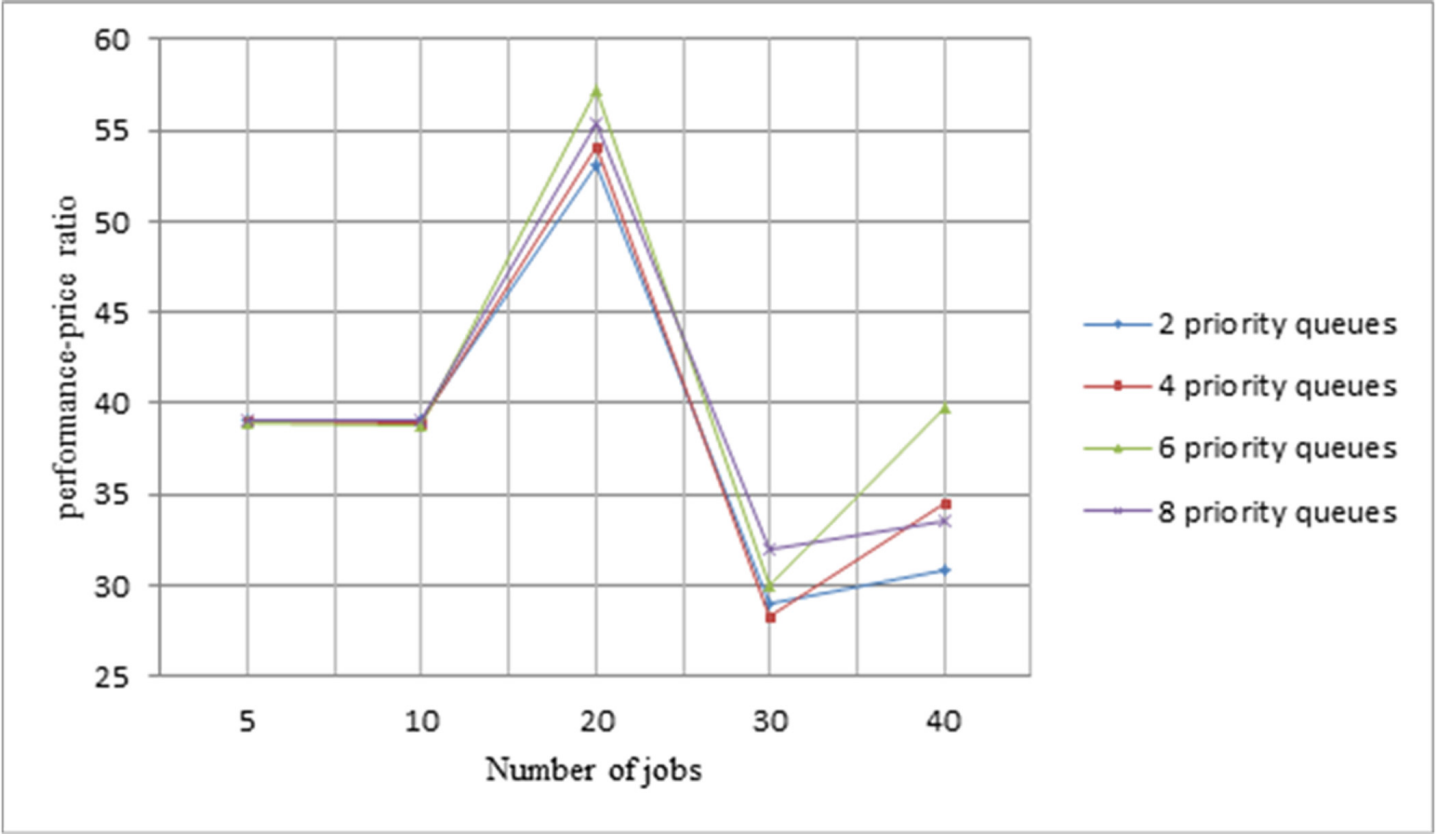
Selecting a suitable number for priority queues. Different numbers of jobs are displayed in x-axis, and y-axis presents the average performance-price ratio of jobs. On the right side, 2, 4, 6, 8 represents different numbers of priority queues.

So as to choose a suitable number for priority queues, we compare the number of priority queues in range of 2, 4, 6, 8, and moreover, every priority queue has capacity to accommodate five jobs. The number of arriving jobs is within 5, 10, 20, 30 and 40. In addition, according to the second section of experiments, we select task number is 32 and helper number is 8, which present the shortest execution time and lowest cost when the number of scheduling times is 30. The average performance-price ratio of jobs in different numbers of priority queues is shown in Fig 9.

The lines in Fig 9 show a tendency from rise to decline to rise of performance-price ratio of different number of jobs in priority queues. The performance-price ratio remains stable when the jobs’ number is in the range of five to ten, and then, when there are twenty jobs arrive simultaneously, the highest performance-price ratio emerges no matter how many priority queues. Moreover, if 30 jobs reach cloudlet at the same time, even though the priority number is different, they all present the lowest performance-price ratio. However, with the increasing number of jobs, average performance-price ration starts to recover. We could find that the green line, which means six priority queues, appears a better trend, and the performance-price ratios of jobs in it are higher than 35 except 30 jobs reach at the same time. When there are 20 or 40 jobs in the system, it even presents the best ratio. Therefore, in the following experiments, we choose the number of priority queues is six.

#### 3.2 Experiments Comparing *LMCpri* and *LMCque*

In order to present all of possible situations, we choose the number of jobs in 5, 10, 20, 30, 40 again. These two frameworks measure the same jobs to control variable. Moreover, the number of priority queues is 6 and the storage capability of every priority queue is five jobs. The same as previous experiment, the task number is 32 and the helper number is eight.

**Fig 10:**
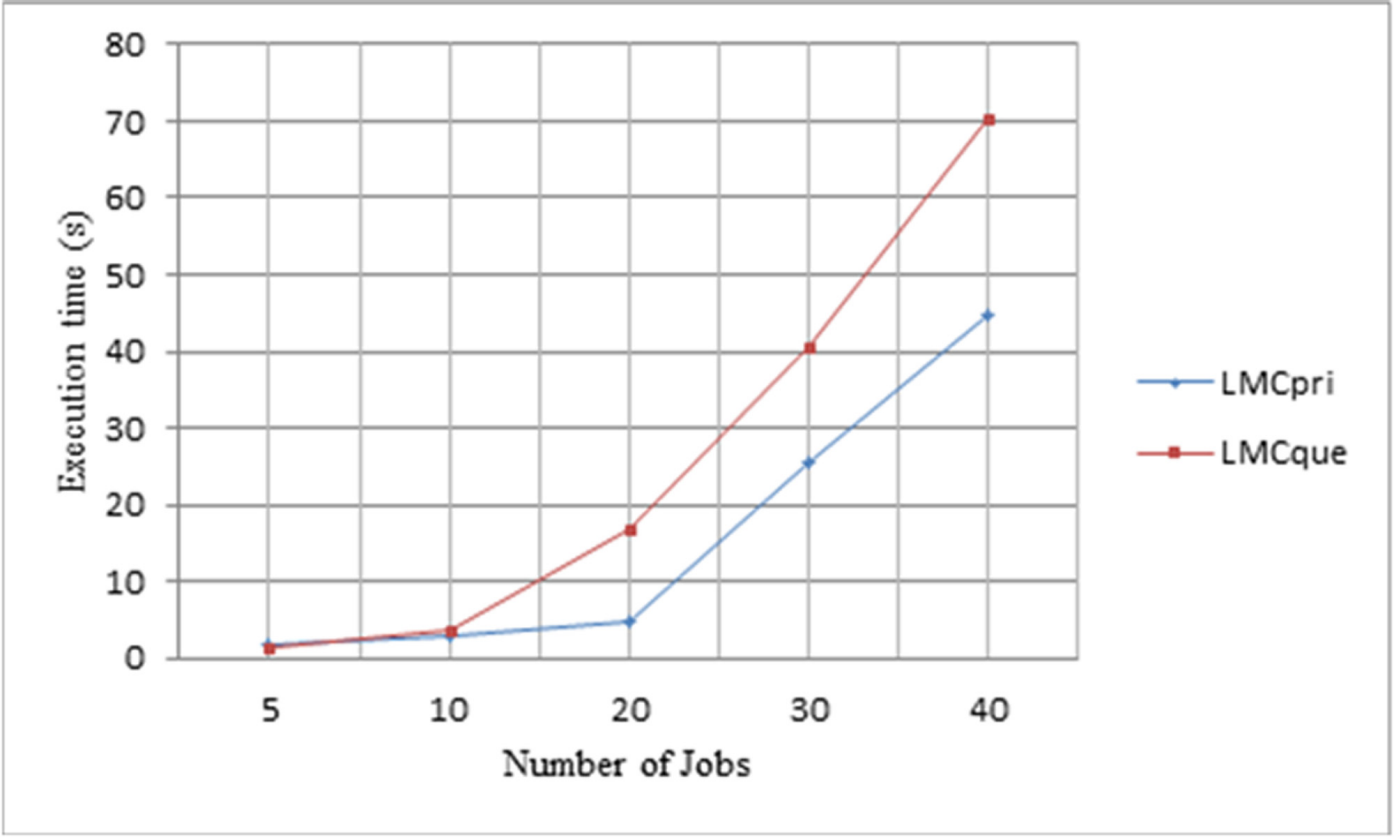
Comparing *LMCpri* with *LMCque* at execution time. Different numbers of jobs are displayed in x-axis, and y-axis presents the average execution time of jobs.

As Fig 10 displays, jobs in *LMCpri* present a shorter execution time than *LMCque* in general tendency. When there are not so many jobs coming simultaneously, the lines of their processing time overlap with each other. That is because jobs’ number is few, the current best position in priority queues does not plays its effect fully. With the number of jobs increasing, *LMCpri* presents its advantages gradually. When there are 40 jobs arrive at the same time, average execution time of jobs in *LMCpri* remains lower than 60s, but in *LMCque*, it has raised more than one minute. We discover that even though auction process of priority queues introduces extra time, the strategy of choosing the current best position for winner still saves more execution time. Therefore, *LMCpri* shows a better performance than *LMCque* no matter the number of arriving jobs.

### 4. Comparing *LMCpri* and Cloud Center Architecture

In this section, we utilize CloudSim platform [23] to simulate cloud datacenter and make a comparison between *LMCpri* and cloud assisting architecture (*CAA*) through the gradually increasing number of jobs, which are defined by 5, 10, 20, 30, respectively, in every group of contrast test. In the experiments, we modify the number of helpers and tasks to testify comprehensively. In order to uniform variants in the two mobile cloud architectures, the number of helpers equals to the number of VMs in CloudSim, and similarly, the number of tasks is the same in both two architectures.

**Fig 11:**
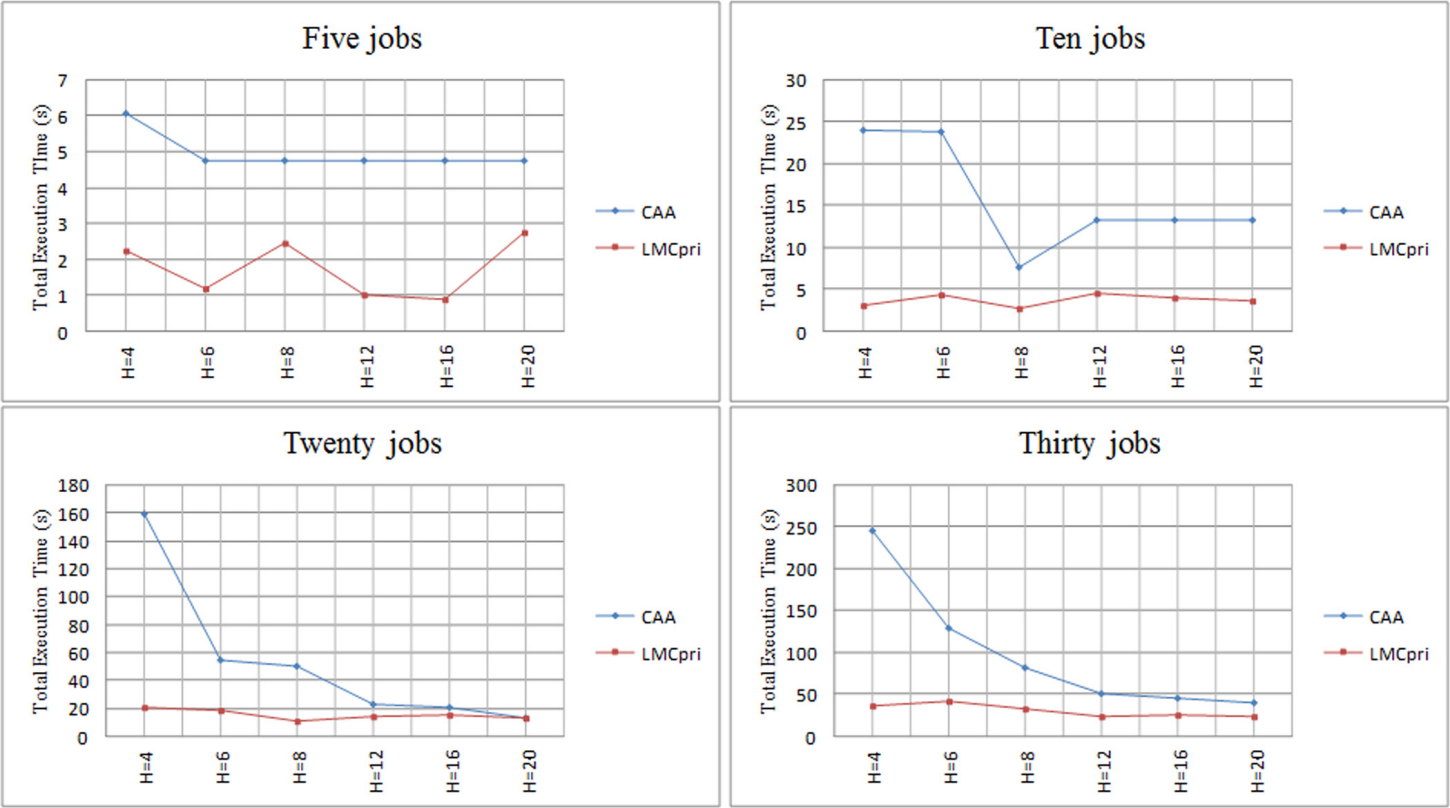
Comparing total execution time of LMCpri and cloud assisting architecture (CAA). *H* means the number of helpers in x-axis.

As Fig 11 shows, with the increasing number of jobs, both *LMCpri* and *CAA* present a raising execution time tendency. When there are five jobs, the longest total execution time of *LMCpri* and *CAA* are 2.75s and 6.08s, respectively, while when there are twenty jobs, the longest time of *LMCpri* and *CAA* are 41.45s and near 250s, respectively. In addition, we can clearly find that following the augment of job number, the lines of execution time of *LMCpri* and *CAA* coincide gradually. This is because cloud centers are designed for large scale applications, and present excellent performance. While there are not so many jobs due to *LAN*, therefore, our architecture displays greater performance than cloud assisting architecture. Moreover, we find that there is a plateau in the first and second figures, and that is because jobs are not split in CloudSim platform, every job is scheduled as an entirety. If they were split into tasks, it would augment workload of scheduling procedure, and bring plenty of extra time.

## Conclusion and Future Work

In this paper, we exhibit a compounded local mobile cloud computing architecture with dynamic priority queues (*LMCpri*) to achieve high QoS of requesters. The architecture should meet minimum execution time and cost of requesters, and we give its main idea, simulation implementation and evaluation. In addition, we are focused on the scheduling algorithm in cloudlet, and NSGA-II is chosen as scheduling algorithm through comparing with PSO and sequential scheduling. In order to satisfy the business model and save execution time, the number of iteration times is confirmed as 30. So as to choose the best number of priority queues of *LMCpri*, performance-price ratio is introduced, which is calculated by *lifeTime*, execution time and the total cost, and we prove the most suitable number is six through experiments. Comparing with *LMCque*, *LMCpri* has a short execution time no matter jobs’ number. Finally, after comparing it with cloud assisting architecture through *CloudSim* platform, *LMCpri* is approved more suitable than cloud assisting architecture in *LAN*, since it takes shorter execution time when there are not so many jobs and helpers simultaneously.

In future work, we plan to thoroughly improve the architecture. Firstly, we will seek to achieve the architecture on hardware, not only simulation. Secondly, the partitioning and scheduling algorithms will be selected strictly to present a better performance than now. Finally, appropriate employment rules will be proposed which is used to solve the problem that many cloudlet response to the same requester at the same time.
